# Altered EEG-derived functional brain states in Parkinson's disease with mild cognitive impairment

**DOI:** 10.1093/braincomms/fcag348

**Published:** 2026-09-11

**Authors:** Yuxin Wang, Zhen Zhang, Jiang Wang, Zhiqi Jiang, Lifang She, Yang Pan, Chen Liu

**Affiliations:** School of Electrical and Information Engineering, Tianjin University, Tianjin 300072, China; School of Electrical and Information Engineering, Tianjin University, Tianjin 300072, China; School of Electrical and Information Engineering, Tianjin University, Tianjin 300072, China; School of Electrical and Information Engineering, Tianjin University, Tianjin 300072, China; Department of Geriatrics, The Affiliated Brain Hospital of Nanjing Medical University, Nanjing, Jiangsu 210029, China; Department of Geriatrics, The Affiliated Brain Hospital of Nanjing Medical University, Nanjing, Jiangsu 210029, China; Department of Neurology, Zhongnan Hospital of Wuhan University, Wuhan, Hubei 430071, China; School of Electrical and Information Engineering, Tianjin University, Tianjin 300072, China

**Keywords:** EEG source imaging, leading eigenvector dynamics analysis, mild cognitive impairment, dynamic feature analysis

## Abstract

The neurobiological mechanism underpinning cognitive impairment in Parkinson's disease remains poorly understood, although it increasingly points to aberrant spatiotemporal switching among large-scale network configurations. Leveraging the high temporal resolution of source-reconstructed EEG, we characterized these microstate alterations and their direct coupling with cognitive performance. This study used an enhanced Leading eigenvector dynamics analysis framework on source-reconstructed EEG data to probe the temporal properties and transition dynamics of brain functional states in Parkinson's disease patients with and without mild cognitive impairment. EEG signals were projected into source space via individual MRI-constrained source imaging, producing time series from 400 cortical regions. Recurring brain states were identified based on instantaneous phase coherence. We computed each state occupancy rate, state duration, and transition probabilities in both Parkinson's disease patients with and without mild cognitive impairment groups. Correlations with Montreal Cognitive Assessment scores were assessed. By identifying four recurrent brain states (states A–D), Parkinson's disease patients with mild cognitive impairment exhibited increased state occupancy rate and state duration in state B and decreased engagement in state D. In addition, transitions into state D from other states were significantly diminished in the mild cognitive impairment group. Crucially, both the temporal expression and kinetic accessibility of state D were positively correlated with Montreal Cognitive Assessment scores, suggesting that this integrated configuration underpins high-order cognitive function and may serve as a neural marker of cognitive resilience in Parkinson's disease. Cognitive deterioration in Parkinson's disease is fundamentally associated with disrupted brain state dynamics, marked by a pathological hyper-convergence towards state B and a systemic attrition of state D. These alterations likely reflect impaired attentional control and network rigidity, and may serve as dynamic biomarkers and therapeutic targets for Parkinson's disease-related cognitive decline.

## Teaser

Parkinson's disease patients with cognitive impairment exhibit altered brain network dynamics. Using EEG, Wang *et al*. found increased duration in a specific brain state and reduced access to another. These disruptions correlate with cognitive scores, suggesting that brain state transitions may serve as biomarkers for cognitive decline.

## Graphical Abstract

**Graphical Abstract:**
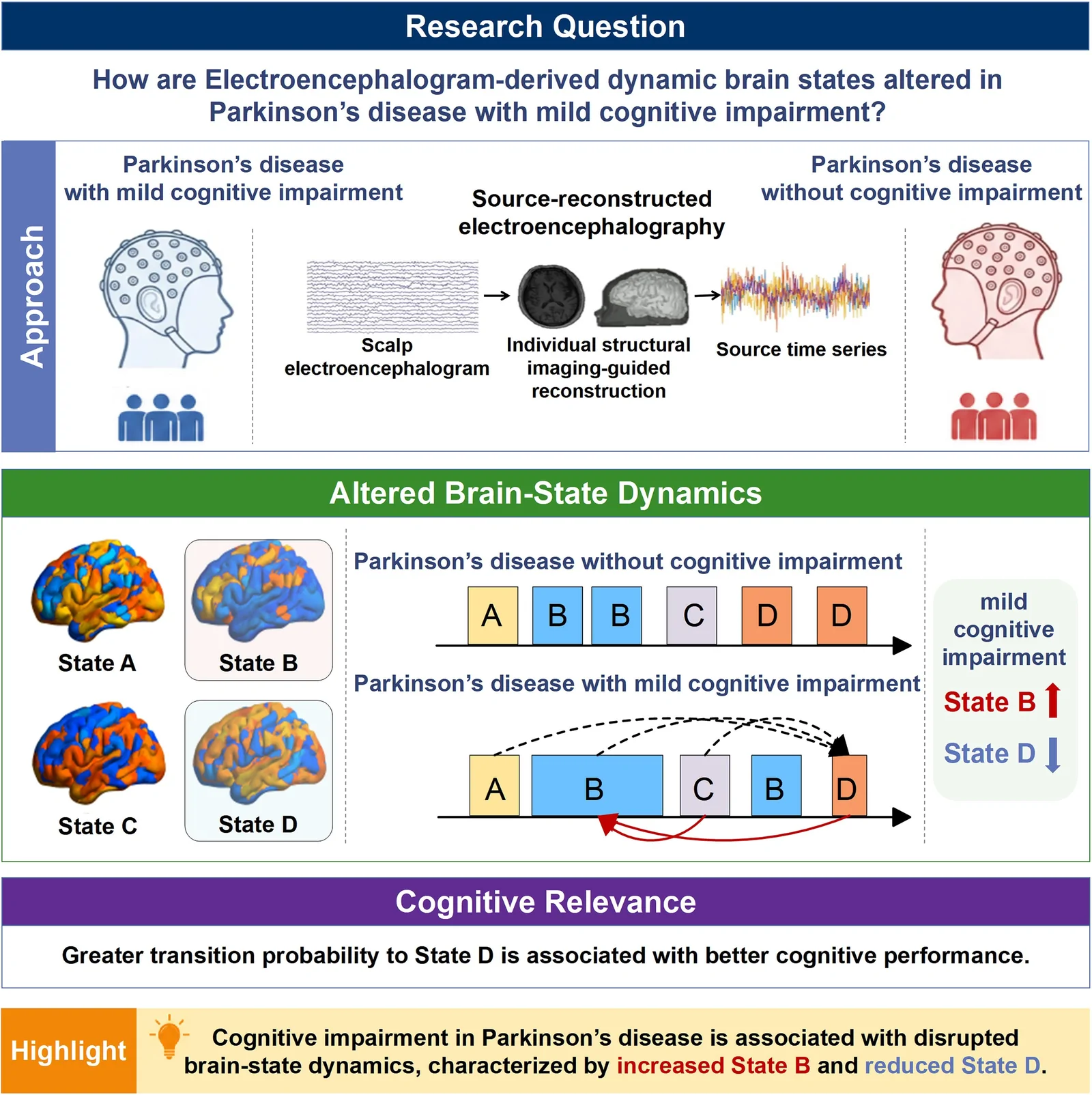

## Introduction

The human brain is a highly dynamic, high-dimensional system that integrates information from different regions to support cognitive function. Resting-state networks (RSNs), which represent spontaneous, functionally meaningful activations of spatially distributed brain regions during rest, have been extensively studied to illuminate their underlying generative mechanisms and functional roles.^1,2^ Characterizing the temporal evolution of these spatially coherent functional patterns is crucial for gaining deeper insights into brain function, particularly in the context of neurological disorders such as Parkinson's disease.^3^

Recent research has prioritized the characterization of how these spatially coherent RSNs dynamically evolve over time, highlighting the importance of capturing complex brain dynamics.^4,5^ Extending our prior study of dynamic brain networks,^6^ we here interrogate how specific brain regions co-activate within these evolving networks, a question that remains insufficiently explored. A commonly used approach to studying dynamic functional connectivity (dFC) is the sliding-window method, which involves segmenting resting-state time series from different brain regions into shorter subsets (windows) and estimating functional connectivity within each window.^7^ However, this method has notable limitations in temporal resolution and statistical reliability. The selection of window length and step size inevitably biases statistical outcomes, making the results highly sensitive to parameter choices.^8^ To overcome these limitations, alternative approaches such as single-frame functional connectivity analysis have been proposed.

Single-frame connectivity methods consider either co-activation patterns (CAPs) or phase coherence approaches such as Leading Eigenvector Dynamic Analysis (LEiDA) to characterize instantaneous brain activity.^9-12^ CAPs detect brief epochs of high-amplitude co-activation and cluster these into recurring spatial patterns. However, applying CAPs to EEG data presents several challenges: EEG signals are inherently noisy and volume-conducted, which complicates the accurate identification of source-level co-activations. Moreover, CAPs traditionally rely on amplitude thresholds, which may be less directly applicable to EEG where phase synchronization often plays a critical functional role.^11,13^ In contrast, LEiDA captures instantaneous relationships between brain regions via relative phase alignment, eliminating the need for thresholding and enabling sensitivity to phase-shifted interactions.^11^ LEiDA represents instantaneous functional connectivity by extracting the leading eigenvector of the phase synchrony matrix at each time point. This method reduces dimensionality while retaining dominant connectivity patterns and allows for the identification of recurring brain states through clustering.^12^ By clustering these leading eigenvectors over time, LEiDA identifies discrete, recurring brain states whose fractional occupancy, transition probabilities, and state duration summarize dynamic brain activity. LEiDA has demonstrated robustness in capturing functional connectivity dynamics and its overlap with known RSNs, making it a promising tool for exploring brain network alterations in neurological disorders.

Despite its broad applicability and conceptual strengths, LEiDA is constrained by two main limitations. First, reliance on a single dominant eigenvector can restrict its capacity to represent complex, multimodal brain states. Additionally, while LEiDA has been validated primarily in functional MRI (fMRI) studies with good results, its performance on electrophysiological data such as EEG or magnetoencephalography (MEG) remains suboptimal due to heightened sensitivity to high-frequency noise and the methodological challenges posed by the high temporal resolution of these signals.^13^ To address these challenges, we developed an enhanced LEiDA framework optimized to overcome its limitations in analysing high-temporal-resolution EEG signals and improve its capability in characterizing dynamic brain networks more comprehensively. When applied to source-reconstructed EEG, this framework improves spatial specificity and permits individualized modelling based on subject anatomy.

Understanding the temporal and spatial variability of dynamic brain networks is particularly relevant for studying Parkinson’s disease, a neurodegenerative disorder characterized by motor and cognitive impairments.^14,15^ The human brain operates as a spatiotemporally interactive network, exhibiting significant variability in both functional and structural organization. However, research on Parkinson’s disease–related resting-state brain network alterations has rarely addressed the spatiotemporal properties of dynamic brain networks. Specifically, variations in brain network dynamics related to specific brain regions and their spatiotemporal characteristics remain underexplored. Since LEiDA captures both temporal and spatial characteristics of brain activity, an improved LEiDA-based approach could provide critical insights into Parkinson’s disease-related network alterations.

Here, we use the enhanced LEiDA framework to characterize the dynamic functional network properties of Parkinson’s disease patients with and without mild cognitive impairment (MCI). Using source-reconstructed neuroimaging signals, we identify brain functional states based on regional connectivity patterns and assess differences in brain state dynamics among Parkinson’s disease patients with varying cognitive levels. We examine the relationship between brain state dynamics and clinical cognitive scores to elucidate how Parkinson’s disease-induced network alterations reflect cognitive dysfunction. By refining the LEiDA methodology and applying it to Parkinson’s disease with MCI, this study aimed to provide novel insights into the pathological mechanisms underlying Parkinson’s disease-related cognitive deficits and improve the characterization of brain network dynamics in neurodegenerative diseases.

## Materials and methods

### Participants

This study included 29 patients with primary Parkinson’s disease, all of whom were screened and enrolled at the Department of Neurology, Nanjing Brain Hospital. Among them, there were 16 males and 13 females, with an age distribution of 68.2 ± 6.8 years (mean ± standard deviation). All participants were examined by the same neurologist according to a unified standard, and their disease severity was confirmed using the Hoehn and Yahr (H-Y) scale to be in stages 1.5–3. The inclusion criteria for participants were as follows: (i) meeting the International Parkinson Disease Association clinical diagnostic criteria for primary Parkinson’s disease^16^; (ii) no head tremor symptoms; (iii) strict adherence to a washout period of at least 12 h before enrolment for anti-Parkinsonian drugs; (iv) no severe hearing impairment to ensure the validity of neuropsychological assessments; and (v) no history of severe head trauma or organic neurological diseases. All participants underwent systematic neuropsychological evaluations, using the Montreal Cognitive Assessment (MoCA)^17^ as the quantitative measure of cognitive function. A total score below 23 indicated cognitive impairment,^18^ and participants were divided into two groups based on this criterion: Parkinson’s disease with mild cognitive impairment and Parkinson’s disease without cognitive impairment (nMCI). Detailed participant information is provided in Table 1. This study strictly follows the ethical guidelines of the Helsinki Declaration.^19^ The research protocol was approved by the Medical Ethics Committee of Nanjing Brain Hospital. All subjects signed written informed consent and fully knew the purpose of the study and the data use standards.

**Table 1:** Basic information about Parkinson’s disease with MCI and nMCI patients

	Parkinson’s disease with MCI mean ± SD	Parkinson’s disease with nMCI mean ± SD	Statistical test result
Gender (male/female)	7/6	9/7	χ2=0.017 ; *P* = 0.897
Age	69.1 ± 6.8	67.5 ± 7.0	t = −0.615; *P* = 0.544
H-Y	2.2 ± 0.5	2.3 ± 0.5	t = 0.280; *P* = 0.782
MoCA scores	16.6 ± 3.1	24.6 ± 2.4	t = 7.879; *P* < 0.001

H-Y, Hoehn-Yahr scale; MCI, mild cognitive impairment; MoCA, the Montreal Cognitive Assessment; nMCI, without mild cognitive impairment, SD, standard deviation.

### Acquisition protocol

Scalp EEG signals in this study were recorded using the Geodesic EEG System 400 (GES 400; Electrical Geodesics Inc., USA). The system was equipped with 64 Ag/AgCl electrodes positioned according to the international 10–10 system. During data acquisition, participants were seated in a quiet, dimly lit room, instructed to keep their eyes closed and remain relaxed throughout the session. Conductive gel was applied to each electrode cup using a blunt syringe to ensure stable contact between the scalp and electrodes, with impedance maintained less than 10 kΩ. EEG signals were sampled at a frequency of 1024 Hz, and the signal quality was continuously monitored by trained personnel. Each recording session lasted approximately 15 ± 2 min. Upon completion of EEG acquisition, the same physician administered the Montreal Cognitive Assessment (MoCA) following a standardized protocol, covering seven cognitive domains: visuospatial and executive functions, naming, attention, language, abstraction, memory, and orientation.

Structural image data were acquired using a Siemens 3.0T Verio MRI scanner (Siemens Healthineers, Erlangen, Germany) with an 8-channel radiofrequency head coil. A high-resolution T1-weighted image was obtained using a three-dimensional magnetization-prepared rapid gradient echo (3D-MPRAGE) sequence. Key acquisition parameters were as follows: repetition time (TR) = 1900 ms, echo time (TE) = 2.48 ms, inversion time (TI) = 900 ms, flip angle = 9°, matrix size = 256 × 256, field of view (FOV) = 250 × 250 mm^2^, slice thickness = 1 mm with no gap, 176 sagittal slices acquired, and voxel size = 1 × 1 × 1 mm^3^. The total scan time was 4 min and 18 s.

#### EEG preprocessing

EEG data preprocessing was performed using the EEGLAB toolbox on the raw 64-channel recordings. A band-pass filter ranging from 1 to 40 Hz was applied to remove noise outside the physiological frequency bands. Zero-phase filtering was conducted using a finite impulse response (FIR) filter with linear phase characteristics to preserve the temporal structure of the EEG signal. After filtering, the data were downsampled to 250 Hz to reduce storage demands and improve computational efficiency while maintaining adequate temporal resolution.

To eliminate spatial bias introduced by monopolar referencing, an average reference re-referencing procedure was implemented. Specifically, the mean voltage across all valid electrodes at each time point was computed and subtracted from each channel, resulting in a differential signal relative to the global average. This re-referencing approach mitigates the effects of volume conduction and enhances cross-subject comparability of network-level features.

Independent component analysis^20^ (ICA) was then applied for blind source separation and artefact rejection.^21^ The procedure involved the following steps: (i) the extended Infomax algorithm was used via the runica function in EEGLAB to decompose the EEG signals into independent components; (ii) the ICLabel^22^ plugin's automated classifier was employed to estimate the correlation of each component with canonical electrooculogram (EOG) and electromyogram (EMG) templates, and components with a correlation coefficient greater than 0.85 were rejected; (iii) manual inspection was conducted to identify and mark residual artefact components; and (4) the identified artefact-related components were set to zero and excluded from signal reconstruction.

Despite the use of automated artefact rejection algorithms, transient high-amplitude noise induced by involuntary movements such as head shifts or swallowing may still remain. These artefact segments often exceed ±80 μV, which falls outside the typical dynamic range of physiological EEG oscillations (−80 μV to +80 μV). Due to their nonstationary temporal characteristics and spectral overlap with neural activity, such artefacts are difficult to eliminate entirely through ICA or band-pass filtering alone. Therefore, a final visual inspection was conducted to validate signal quality.

Using EEGLAB's visual inspection tools, the entire EEG time series was reviewed in 10-second windows. Segments exhibiting amplitudes exceeding ±100 μV or with sustained low variance (<0.5 μV^2^) were excluded. After preprocessing, 180 s of artefact-free EEG data were retained for each subject for subsequent analysis.

### EEG source imaging

EEG source imaging, as a core non-invasive technique for localizing neural electrical activity, solves the electromagnetic inverse problem to project scalp-recorded potentials onto the cortical source space, thereby enabling high spatiotemporal resolution analysis of brain network dynamics.^23^ The source reconstruction process consists of two key components: the forward modelling and the inverse problem solution.

The forward problem involves computing the theoretical scalp potential distribution generated by known current sources within the brain, based on Maxwell's equations and a realistic head model. In contrast, the inverse problem seeks to estimate the unknown parameters of brain sources—including their location, orientation, and amplitude—based on observed scalp potentials. Given that the number of scalp electrodes is substantially lower than the number of potential source locations, the EEG inverse problem is inherently ill-posed and underdetermined. Thus, physiologically meaningful solutions require the imposition of structural constraints and the application of appropriate regularization techniques.

In this study, source localization was conducted using the open-source MATLAB toolbox Brainstorm,^24^ applied to clinical 64-channel EEG recordings. The goal was to estimate the cortical distribution of neural activity underlying the observed scalp EEG signals.

#### Head model construction

Individualized head models were constructed using each participant's structural MRI data, as illustrated in Fig. 1. First, preprocessing of MRI images was performed using the CAT12 toolbox,^25^ including bias field correction to minimize greyscale intensity drift caused by magnetic field inhomogeneities, and skull stripping to remove non-brain tissues such as scalp and skull, thereby isolating intracranial structures and enabling accurate delineation of brain tissue boundaries for subsequent segmentation.

**Figure 1:**
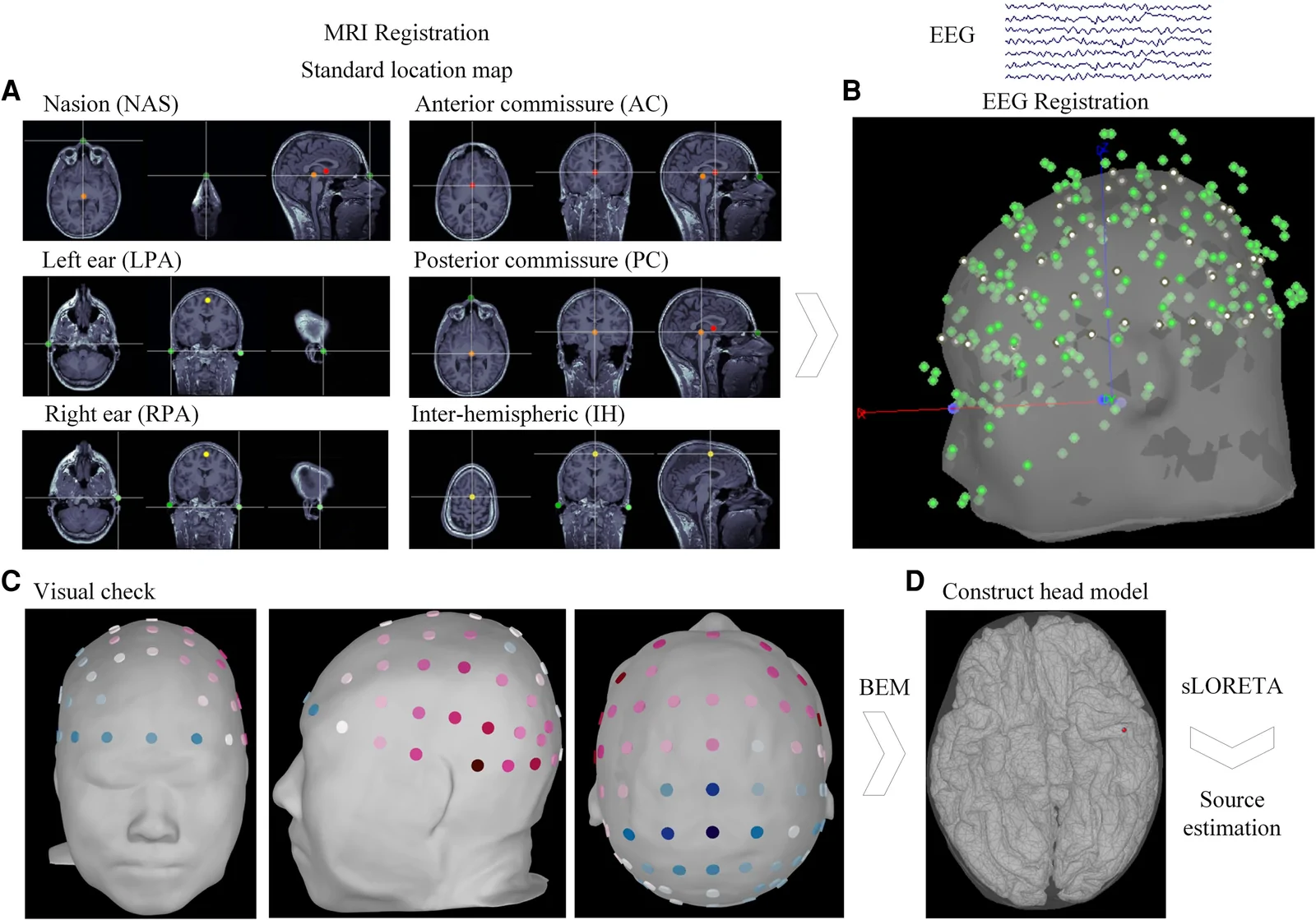
**Pipeline for EEG source localization and head model construction.** EEG signals were projected into cortical source space using individualized MRI-constrained forward models and an inverse solution based on standardized low-resolution brain electromagnetic tomography. Time series were extracted from 400 cortical regions of interest defined by a high-resolution parcellation scheme. (**A**) MRI registration using standard anatomical landmarks. (**B**) EEG registration: mapping of EEG electrode positions onto a 3D head model. (**C**) Visual check of electrode placement on the head surface from multiple views. (**D**) Head model construction using BEM and source estimation using sLORETA. AC, anterior commissure; BEM, boundary element method; IH, inter-hemispheric; LPA, left ear; NAS, Nasion; PA, posterior commissure; RPA, right ear; sLORETA, standardized low-resolution brain electromagnetic tomography.

Next, brain tissue segmentation was carried out using an adaptive maximum a posteriori (AMAP) approach. This method incorporates anatomical priors and applies fuzzy classification to estimate the probability of each voxel belonging to grey matter (GM), white matter (WM), or cerebrospinal fluid (CSF). Compared to conventional Gaussian mixture model (GMM)-based segmentation techniques, the AMAP approach exhibits better adaptability to individual anatomical variability and improves segmentation accuracy.

Following segmentation, each participant's MRI was registered to the MNI standard space using an affine transformation estimated by SPM mutual information-based algorithm (maff8). This registration computed a 4 × 4 affine matrix that aligned the subject's MRI with the MNI template, ensuring inter-subject comparability while preserving the overall anatomical integrity of the brain. Subsequently, cortical surface reconstruction was performed. The inner (WM-GM interface) and outer (GM-CSF interface) surfaces were extracted to generate a high-resolution cortical mesh, which defined the boundary of the source space. This cortical mesh constrained the source space of the EEG inverse solution, ensuring anatomical plausibility in source localization.

Finally, a three-layer realistic head model was constructed using the boundary element method (BEM).^26^ The surfaces of the scalp, outer skull, and inner skull were discretized into meshes, each with 1922 vertices, allowing accurate modelling of electrical field propagation across tissue boundaries. The BEM assumes homogeneous conductivity within each compartment and solves the forward problem by modelling the potential on these boundary surfaces, achieving a balance between computational efficiency and accuracy.

The BEM head model was integrated with individual MRIs using the OpenEEG interface,^27^ generating subject-specific head models that accurately reflect individual anatomical features. These models provide the leadfield matrix, which defines the forward mapping between cortical source activity and scalp-recorded EEG signals. Ultimately, the relationship between the observed EEG potentials and the underlying source currents can be described as:


(1) Φ=KJ+cl

where *Φ* is an *N*-dimensional vector representing the scalp EEG potentials, and J denotes the distributed source current density. The scalar *c* is a constant, and l is an *N*-dimensional unit vector. K is the leadfield matrix obtained from the forward model, which characterizes the relationship between source activity within the brain and the observed signals at the scalp electrodes. This modelling framework enables a more accurate description of the propagation of EEG signals through different head tissues, thereby enhancing the precision and physiological plausibility of EEG source localization analysis.

#### Source space signal estimation and extraction

The core of EEG source localization lies in constructing a physiologically realistic source model that describes the spatial distribution of intracranial current generators and solving the corresponding inverse problem. In this study, a distributed source model was adopted, in which the cortical surface was discretized into a high-resolution mesh consisting of 15 000 vertices. Neural electrical activity at each node of the mesh was modelled as a current dipole, and the inverse problem was formulated as the estimation of the dipole orientation and magnitude at each node.

To better capture the complexity of brain activity, the dipole orientations were set as unconstrained, allowing current vectors at each source point to vary freely in three-dimensional space rather than being fixed along the cortical normal. To characterize the background noise properties of the EEG signals, the sensor-level noise covariance matrix was computed from resting-state EEG recordings. This noise covariance matrix served as a regularization term in the inverse solution, refining the minimum norm estimates and improving the spatial accuracy of source localization.

The inverse problem was solved using standardized low-resolution brain electromagnetic tomography (sLORETA),^28^ a method derived from minimum norm imaging (MNI). sLORETA introduces a standardization constraint into the cost function minimization (Eq. 2), which effectively reduces the superficial source bias inherent in conventional MNI approaches and improves the spatial resolution and localization accuracy of deep sources.


(2) $$\underset{J,c}{\min F}=\|\Phi -KJ-cl{\|}^{2}+\alpha \|J{\|}^{2}$$

where *α* is the regularization parameter. Diagonal noise covariance regularization was employed to enhance the robustness of the solution and mitigate the impact of unstable estimates.

After the computation of source-level signals, time series were further extracted from regions of interest (ROI) to investigate the activity patterns across different brain areas. In this study, the cortical surface was parcellated using the Schaefer atlas with 400 functionally defined ROIs. Source signals were aggregated at the ROI level by averaging the time courses of all vertices within each region, yielding a representative mean time series for each brain area. These ROI-based time series were subsequently used for functional brain state analyses.

### Improved LEiDA algorithm

The functional connectivity between different brain regions is not static but exhibits complex temporal dynamics. Therefore, an effective analytical method is required to capture these variations. LEiDA algorithm, proposed by Cabral *et al*.,^12^ aims to identify the instantaneous functional connectivity states of the brain by extracting the leading eigenvector of the functional connectivity matrix. Unlike traditional functional connectivity analysis methods that operate on two-dimensional connectivity matrices, LEiDA dynamically characterizes the evolution of brain connectivity patterns over time using a series of one-dimensional eigenvectors (i.e. phase-locking patterns), thereby improving computational efficiency and clustering performance. The method primarily describes the dynamic functional states of brain networks by extracting the leading eigenvector of the instantaneous phase coherence matrix derived from time-series signals.

The implementation of LEiDA consists of several steps,^29^ the flow chart is shown in Figs 2 and 3. First, to extract the phase information of the signals, the Hilbert transform is applied to the time-series signal of each ROI *n* (*n* = 1 … *N*, where *N* is the total number of ROIs), as defined in Eq. 3. The first and last time points are removed from each time-series signal to eliminate boundary artefacts introduced by the Hilbert transform.


(3) $$H(x(t))=x(t)+i\cdot \hat{x}(t)$$

where x(t) is the original signal, $\hat{x}(t)$ represents the Hilbert transform-derived orthogonal component, and H(x(t)) is the analytic signal. Using the complex-valued signal, the instantaneous phase can be computed as:


(4) θ(t)=arctan(Im(H(x(t)))Re(H(x(t))))=arctan(x^(t)x(t))

**Figure 2:**
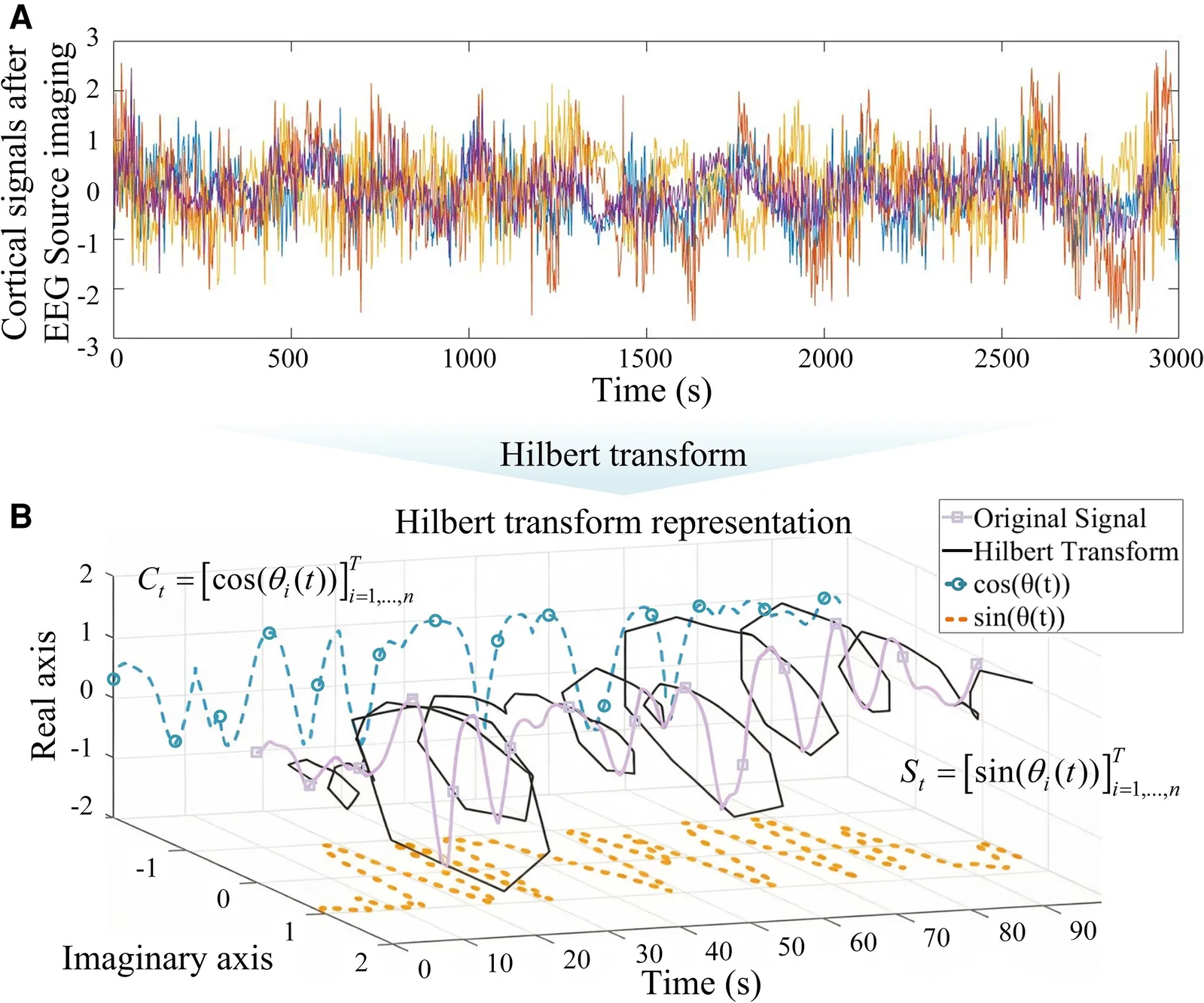
**Hilbert transform representation of EEG source signals.** (**A**) EEG-derived cortical time series for multiple sources over a 3000-second recording period. (**B**) Hilbert transform representation in the complex plane, showing the projection of the original signal as a solid line with square markers and its Hilbert transform as a solid line. The corresponding cosine and sine components are shown as a dashed line with circle markers and a dashed line without any markers, respectively.

**Figure 3:**
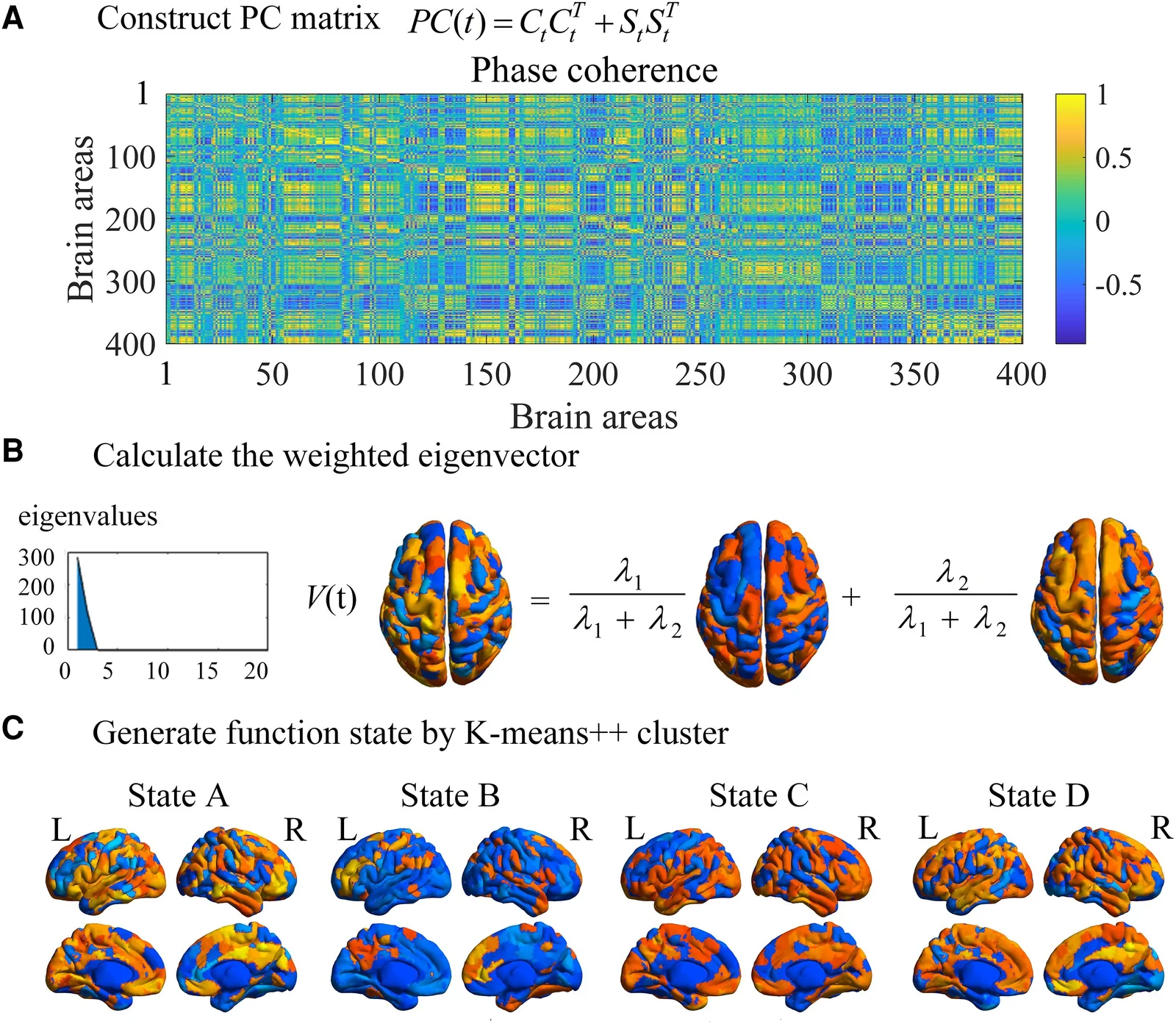
**Flow chart of the improved LEiDA method.** (**A**) Construct phase coherence matrices to characterize inter-regional synchrony; (**B**) Compute the weighted eigenvectors V(t) to capture dominant phase alignment patterns; (**C**) Use k-means++ clustering to identify four distinct brain functional states. L, left; R, right.

Next, the instantaneous phase coherence matrix is computed for each time point *t* based on the obtained instantaneous phase values. The matrix elements are determined by the phase differences between regions *n* and *m*, as shown in Eq. 5. This matrix reflects the instantaneous phase synchronization between different brain regions, where $PC_{nm}(t)=1$ indicates complete synchronization between regions *n* and *m* at time *t*.


(5) $$PC_{ij}(t)=\cos\;(\theta_{i}(t)-\theta_{j}(t))$$

In the LEiDA method, only the leading eigenvector (1 × *N*) of the phase coherence matrix is considered to describe the phase-locking pattern at each time point. Specifically, an eigenvalue decomposition of the phase coherence matrix PC(t) is performed to extract the eigenvector V(t) corresponding to the largest eigenvalue $\lambda_{1}$:


(6) $$PC(t)\cdot V(t)=\lambda_{1}V(t)$$

The leading eigenvector V(t) describes the dominant functional connectivity pattern at time *t*, where each component represents the extent to which a brain region participates in the dominant connectivity pattern. Ultimately, a leading eigenvector is obtained at each time point, which is then used for further analysis of brain functional state dynamics.

Although LEiDA has demonstrated excellent performance in functional connectivity analysis, it has certain limitations when dealing with complex brain network dynamics. First, LEiDA constructs the functional connectivity matrix solely based on instantaneous phase differences, which may not fully capture the intricate phase relationships between brain regions. Second, LEiDA only extracts the eigenvector corresponding to the largest eigenvalue, potentially overlooking crucial information contained in other eigenvectors, resulting in an incomplete description of functional states and increased sensitivity to data noise. To address these limitations, we propose two key improvements:

#### Construction of the phase coherence matrix

After extracting the phase information from time-series signals using the Hilbert transform, the improved method incorporates both cosine and sine components of instantaneous phases to construct a more comprehensive phase coherence matrix, better capturing the dynamic characteristics of cortical signals. Specifically, for each time point *t*, the phase coherence matrix PC(t) is computed as:


(7) $$PC(t)=C_{t}C_{t}^{T}+S_{t}S_{t}^{T}$$

where:


(8) $$C_{t}=[\cos(\theta_{i}(t)){]}_{i=1,\ldots ,n}^{T}$$

(9) $$S_{t}=[\sin\;(\theta_{i}(t)){]}_{i=1,\ldots ,n}^{T}$$

#### Weighted computation of the leading eigenvector

The improved LEiDA method employs an eigenvalue-weighted approach that integrates information from the first two leading eigenvectors, enhancing the representation of functional connectivity states and improving adaptability to different signal environments. Specifically, for the eigenvectors computed from the phase coherence matrix PC(t), a weighted eigenvector V(t) is obtained using the eigenvalues as weights:


(10) $$\begin{array}{rl} V(t)\;= & \frac{\lambda_{1}}{\lambda_{1}+\ldots +\lambda_{i}}V_{1}+\frac{\lambda_{2}}{\lambda_{1}+\ldots +\lambda_{i}}V_{2}+\ldots \\ & +\frac{\lambda_{i}}{\lambda_{1}+\ldots +\lambda_{i}}V_{i} \end{array}$$

Due to the markedly higher magnitude of the two largest eigenvalues compared to the others, we selected *i* = 2, where $\lambda_{1}$ and $\lambda_{2}$ are the two largest eigenvalues, $V_{1}$ and $V_{2}$ are their corresponding eigenvectors.

Ultimately, a weighted eigenvector V(t) is obtained for each time point, representing the phase-locking pattern of brain signals at that moment. Each element of the vector indicates the degree to which a specific brain region participates in the dominant functional connectivity pattern. Consequently, each eigenvector V(t) serves as an observation of the brain's dynamic system, and the evolution of weighted eigenvectors describes the trajectory of dynamic brain states. The weighted combination of multiple eigenvectors enriches the representation of functional connectivity states, enabling a more accurate capture of complex brain network patterns. The weighting scheme reduces sensitivity to noise that might arbitrarily distort a single eigenvector, providing a more stable representation of the dominant phase-locking patterns in EEG. Moreover, this approach reduces the sensitivity of the results to a single eigenvector, enhancing the robustness of the algorithm.

### Clustering brain status by K-means++

To obtain a state-based representation sequence, we applied clustering to the weighted eigenvectors V(t) of all participants, following a similar approach to the conventional LEiDA method. Specifically, we employed the k-means++ clustering algorithm,^30^ which iteratively partitions the dataset of T feature vectors into *K* clusters, minimizing the sum of distances between each data point and its assigned cluster centroid. In this study, the number of clusters *K* was varied from 2 to 12 in increments of 1. For each *K*, the clustering procedure was repeated 100 times, with cluster centroids being reinitialized in each iteration. The clustering result with the lowest error was selected for subsequent analysis.

Compared to the classical k-means algorithm, k-means++ improves the initialization of cluster centroids by selecting them sequentially, ensuring that the next centroid is chosen from data points that are farther from the existing centroids. This strategy reduces the likelihood of instability caused by poor initial centroid selection. The optimal number of states was determined by analysing the silhouette score as a function of the number of clusters, in combination with the elbow method, the optimal number of clusters was determined to be 4.

Finally, all brain regions in the space were assigned to the nearest centroid. That is, each V(t) was represented by its closest centroid, where each centroid corresponds to a phase-locking state. Consequently, the original signal sequence of each participant was transformed into a state-based time series representation.

### Time domain feature analysis

Based on each participant's state sequence, we evaluated several characteristic metrics to quantify the dynamic distribution properties of the four brain functional states. These metrics capture the temporal dynamics of phase-locking states, providing insights into the functional organization of brain activity.

#### State occupancy rate

For each participant *S*, the occupancy rate of a given phase-locking state *α* is calculated using Eq. (11):


(11) $$FO_{\alpha }^{\left(S\right)}=\frac{1}{T}\sum_{t=1}^{T}\chi [S(t)=\alpha ]$$

where *χ* is an indicator function, which χ(A)=1 if the condition *A* is true and χ(A)=0 otherwise. *T* represents the total number of time points in the participant's signal (S). In this study, *T* = 45 000 (corresponding to a scanning duration of 3 min with a sampling frequency of 250 Hz). In other words, state occupancy is the number of times the feature vector V(t) is assigned to a specific cluster divided by the total number of time points *T*. For each participant, we computed the occupancy rates for all four phase-locking states.

#### State duration

The average duration of each visit to the phase-locked state α was calculated from Eq. (12):


(12) $$DT_{\alpha }^{\left(S\right)}=\frac{1}{\;p_{\alpha }}\sum_{1}^{\;p_{\alpha }}C_{\;p_{\alpha }}$$

where $p_{\alpha }$ is the number of consecutive time periods where the phase-locked state α appears, and $C_{\;p_{\alpha }}$ is the duration of each segment.

#### State transition probability

From Eq. (11), the probability $\Pi_{\alpha \beta }$ that the state of time point *t* is α and the state of time point *t* + 1 is β can be expressed as:


(13) $$\Pi_{\alpha \beta }^{\left(S\right)}=\frac{1}{T-1}\sum_{1}^{T-1}\chi [V(t)\in R^{\alpha },V(t+1)\in R^{\beta }]$$

Therefore, the transition probability matrix $W_{\alpha \beta }^{\left(S\right)}$ for each subject can be calculated from Eq. 14:


(14) $$W_{\alpha \beta }^{\left(S\right)}=P[V(t+1)\in R^{\beta }|V(t)\in R^{\alpha }]=\frac{\Pi_{\alpha \beta }^{\left(S\right)}}{\Pi_{\alpha }^{\left(S\right)}}$$

That is, the transition probability matrix from states α to state β, representing the level of dynamic transition between each phase-locked state. For each subject, the transition probability matrix was computed separately.

## Results

### Clustering of brain functional states

The four functional brain states derived via k-means++ clustering characterize the recurrent functional connectivity patterns in cortical brain regions, predicated on the 400 ROIs from the Schaefer cortical segmentation, as shown in Fig. 3(C). To contextualize these recurrent configurations, we mapped a matching alignment of the spatial topologies for each brain state onto the canonical Yeo resting-state brain network.^2^

State A manifests as a robust co-activation of the limbic network (LN) and the default mode network (DMN). The DMN is central to self-referential thought, episodic memory, and mind-wandering, while the LN supports emotional processing and memory consolidation. Co-activation of these networks may reflect introspective or emotionally salient mental states. Importantly, hyperconnectivity within the DMN and its aberrant coupling with limbic regions constitute consistent pathological signatures in Alzheimer's disease, suggesting a potential pathological marker of early neurodegeneration.^31,32^

State B exhibits joint deactivation of the visual network (VN) and LN. This pattern reflects a pathological inability to effectively engage these critical networks. In Parkinson’s disease, the VN is frequently impaired, contributing to visuospatial deficits and hallucinations, while LN dysfunction is phenotypically tied to apathy and emotional blunting.^33^ Metabolic hypofunction and functional silencing in visual and limbic areas are also hallmark features of Alzheimer's disease, particularly in later stages, and have been linked to visuospatial deficits and emotional blunting.^34,35^

State C reflects functional integration among the VN, LN, and frontoparietal networks (FPN). The FPN is critical for executive control, working memory, and goal-directed behaviour. Its integration with visual and limbic regions may facilitate visual attention, emotionally guided executive function, or memory encoding. Similar hyperintegration among FPN, visual, and affective networks has been reported in early Alzheimer's disease, possibly reflecting compensatory mechanisms or pathological network dedifferentiation.^36,37^

Finally, State D is associated with the functional cooperation between the DMN and the ventral attention network (VAN). The VAN supports bottom-up attentional reorienting, and its coupling with the DMN may enable efficient switching between internal thought and external stimuli. Breakdown of VAN-DMN coordination is commonly seen in Alzheimer's disease and mild cognitive impairment, leading to attentional deficits and reduced cognitive flexibility.^38,39^

### Analysis of time domain properties of LEiDA status in Parkinson’s disease with MCI

The quantitative results regarding state occupancy rate and state duration calculations are summarized in Fig. 4 and Table 2. To evaluate inter-group discrepancies in these temporal characteristics between patients with Parkinson’s disease with MCI and nMCI, two-tailed independent samples *t*-tests were conducted, with statistical significance set at *P* < 0.05. Additionally, Spearman correlation analyses were performed to examine the relationship between the significantly altered temporal metrics and the MoCA scores, considering a *P*-value <0.05 as statistically significant.

**Figure 4:**
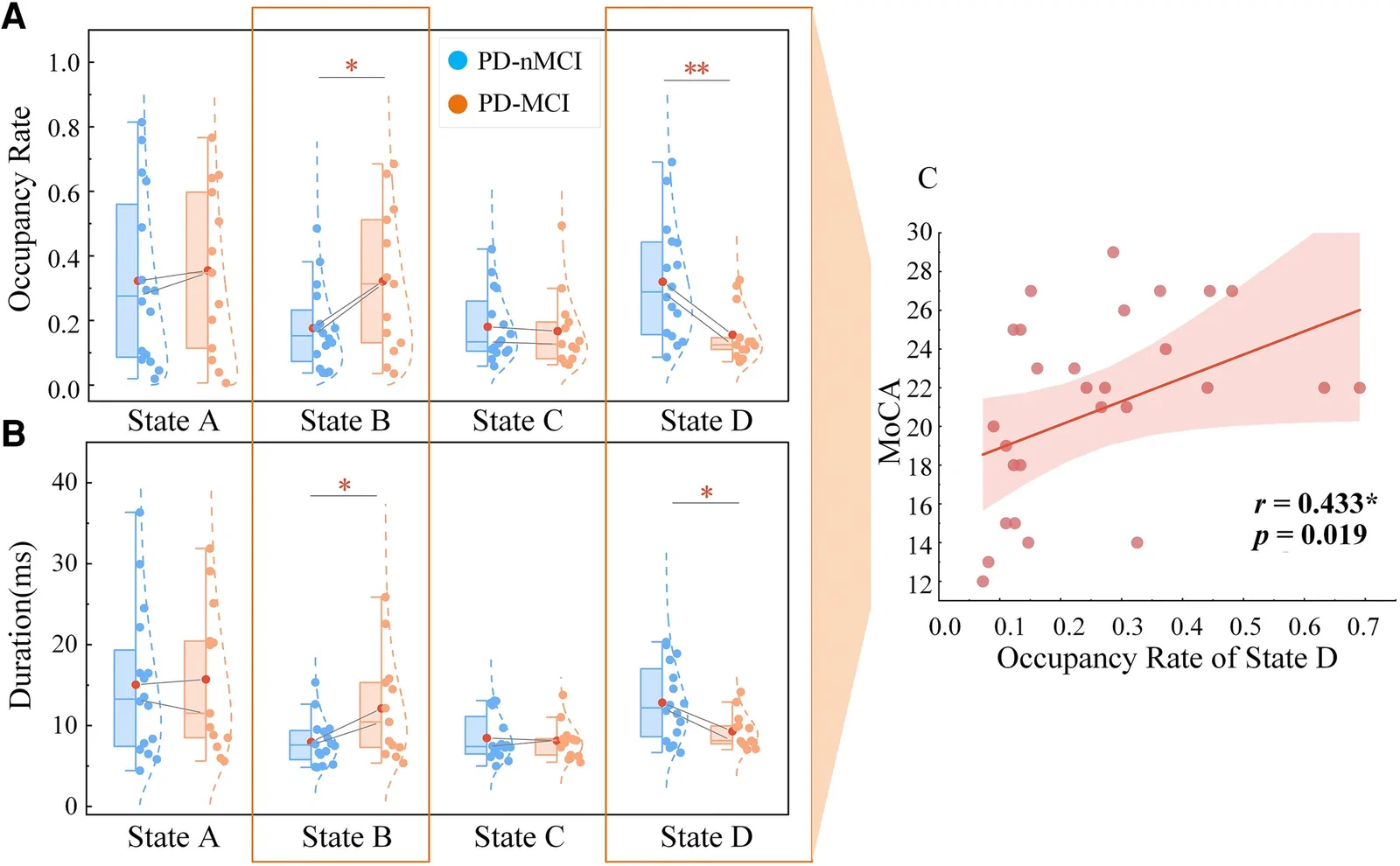
**Statistical results of time characteristic parameters for functional status in Parkinson’s disease with nMCI (*N* = 16) and Parkinson’s disease with MCI (*N* = 13) groups.** (**A**) Box plots showing the independent samples *t*-test results of inter-group differences in state occupancy rate (ms) for each state (A–D) in Parkinson’s disease with nMCI (left) and Parkinson’s disease with MCI (right) groups. Each data point represents the occupancy rate of a specific state for an individual patient; (**B**) Box plots showing the independent samples *t*-test results of inter-group differences in average duration (ms) for each state (A–D) in Parkinson’s disease with nMCI (left) and Parkinson’s disease with MCI (right) groups. Each data point represents the duration of a specific state for an individual patient. Orange boxes highlight states with significant group differences (*P < 0.05); (**C**) The Spearman correlation between state D occupancy rate and MoCA score (*r* = 0.433, *P* = 0.019). Each data point represents the MoCA score and corresponding State D occupancy rate for one patient. Red asterisks indicate the significant results, * For *P* < 0.05, and ** for *P* < 0.01. MCI, mild cognitive impairment; MoCA, Montreal Cognitive Assessment; nMCI, without cognitive impairment. Detailed data are shown in Table 2.

**Table 2:** Parameters of brain functional status and statistical results between Parkinson’s disease with MCI and nMCI groups

Feature parameter	State class	Parkinson’s disease with MCI (Mean/SD)	Parkinson’s disease with nMCI (Mean/SD)	Comparison between groups (*p*/*T*)
Occupancy rate	State A	0.355/0.072	0.323/0.067	*P* = 0.746/*T* = 0.327
	State B	0.321/0.063	0.176/0.032	***P*** **=** **0.039/*T*** **=** **2.171**
	State C	0.167/0.033	0.181/0.027	*P* = 0.755/*T* = −0.316
	State D	0.156/0.024	0.320/0.045	***P*** **=** **0.006/*T*** **=** **−2.990**
Duration	State A	15.719/2.542	15.052/2.296	*P* < 0.847/*T* = 0.195
	State B	12.125/1.797	7.979/0.722	***P*** **<** **0.029/*T*** **=** **2.301**
	State C	8.138/0.624	8.479/0.704	*P* = 0.726/*T* = −0.354
	State D	12.847/0.619	4.665/1.166	***P*** **=** **0.018/*T*** **=** **−2.509**
Transition probability	A-A	0.645/0.057	0.625/0.057	*P* = 0.807/*T* = 0.247
	A-B	0.166/0.035	0.116/0.036	*P* = 0.258/*T* = 1.156
	A-C	0.102/0.024	0.092/0.014	*P* = 0.725/*T* = 0.355
	A-D	0.088/0.013	0.167/0.029	***P*** **=** **0.031/*T*** **=** **−2.281**
	B-A	0.174/0.035	0.178/0.040	*P* = 0.935/*T* = −0.083
	B-B	0.583/0.053	0.443/0.044	*P* = 0.051/*T* = 2.047
	B-C	0.151/0.025	0.189/0.017	*P* = 1.197/*T* = −1.324
	B-D	0.092/0.015	0.189/0.024	***P*** **=** **0.004/*T*** **=** **−3.199**
	C-A	0.171/0.034	0.161/0.037	*P* = 0.849/*T* = 0.192
	C-B	0.245/0.031	0.169/0.019	***P*** **=** **0.040/*T*** **=** **2.157**
	C-C	0.478/0.035	0.483/0.038	*P* = 0.927/*T* = −0.093
	C-D	0.106/0.007	0.187/0.029	***P*** **=** **0.019/*T*** **=** **−2.488**
	D-A	0.169/0.033	0.156/0.035	*P* = 0.795/*T* = 0.263
	D-B	0.164/0.025	0.095/0.013	***P*** **=** **0.014/*T*** **=** **2.616**
	D-C	0.117/0.016	0.103/0.015	*P* = 0.545/*T* = 0.614
	D-D	0.550/0.026	0.646/0.033	***P*** **=** **0.037/*T*** **=** **−2.192**

MCI, mild cognitive impairment; nMCI, without mild cognitive impairment; SD, standard deviation. The bold values indicate that there is a significant difference in characteristics between Parkinson’s disease with MCI and Parkinson’s disease with nMCI groups. The significant results for the occupancy rate and duration are shown in Fig. 4, while the significant results for the transition probabilities are shown in Fig. 5.

The statistical evaluations indicated that the Parkinson’s disease with the MCI group exhibited a significantly elevated occupancy rate (*P* = 0.039, *t* = 2.171) and prolonged state duration (*P* = 0.029, *t* = 2.301) in state B compared to the Parkinson’s disease with the nMCI group. Conversely, the occupancy rate (*P* = 0.006, *t* = −2.990) and state duration (*P* = 0.018, *t* = −2.509) of state D were significantly lower in the Parkinson’s disease with the MCI group. No significant intergroup differences in state occupancy rate or state duration were observed for the other states. These findings suggest that cognitive impairment in Parkinson’s disease patients may be fundamentally associated with a pathological preponderance of state B and a concomitant reduction in the engagement of state D.

Furthermore, the occupancy rate of state D demonstrated a significant positive correlation with the MoCA score (*r* = 0.415, *P* = 0.025), indicating that accelerated cognitive decline (i.e. lower MoCA scores) strongly tracks with a diminished occupancy rate of state D. Collectively, these findings imply that the cognitive status of Parkinson’s disease patients may be intrinsically linked to the expression profiles of states B and D, highlighting the dynamic characteristics of these states hold potential as biomarkers for Parkinson’s disease with MCI.

Crucially, this marked alteration in the macro-scale distribution of brain functional states in Parkinson’s disease with MCI patients clarifies the network-level architecture of cognitive decline, characterized by an increased expression of state B and a decreased expression of state D. Notably, the increased prevalence of state B may reflect abnormal deactivation within the VN and LN, potentially disrupting cognitive control processes associated with these regions. Conversely, the reduced expression of state D suggests dysregulated coordination between the DMN and the VAN, mechanistically pointing to a diminished inhibitory role of the DMN over task-irrelevant networks and resulting in impaired resource allocation. These findings further support the presence of aberrant dynamic functional connectivity in Parkinson’s disease with MCI and suggest that the state occupancy rate and state duration of LEiDA-derived states hold significant clinical utility as potential biomarkers for assessing cognitive decline in Parkinson’s disease.

### LEiDA state dynamic transition properties of Parkinson’s disease with MCI

Table 2 delineates the transition probability matrices across the brain functional states in both the Parkinson’s disease with MCI and nMCI groups, characterizing the dynamic state-switching trajectories tied to cognitive function in Parkinson’s disease. Two-tailed independent samples *t*-test was conducted to evaluate group differences in state transition probabilities, with statistical significance defined at *P* < 0.05. Additionally, Spearman correlation analyses were performed to assess the relationship between dynamic transition properties and the MoCA score, with *P* < 0.05 considered statistically significant.

The results revealed significant alterations in key state transition pathways between the Parkinson’s disease with nMCI and MCI groups, as illustrated in Fig. 5. Compared to the Parkinson’s disease with nMCI group, Parkinson’s disease with MCI patients exhibited a significantly higher probability of transitioning from states C and D to state B (*P* = 0.040, *t* = 2.157; *P* = 0.014, *t* = 2.616), along with a significantly reduced probability of transitioning from all four states into state D (*P* = 0.031, *t* = −2.281; *P* = 0.004, *t* = −3.199; *P* = 0.019, *t* = −2.488; *P* = 0.037, *t* = −2.192). These findings suggest an abnormal dynamic pattern in Parkinson’s disease with MCI characterized by a ‘hyper-convergence toward state B and a systemic attrition away from state D’ transition profile. This kinetic imbalance may reflect a reduced ability of the DMN to suppress task-negative networks (e.g. the VN), potentially leading to a misallocation of cognitive control resources to irrelevant information. This phenomenon aligns with the temporal characteristics observed earlier and further supports the critical roles of states B and D in Parkinson’s disease-related cognitive impairment.

**Figure 5:**
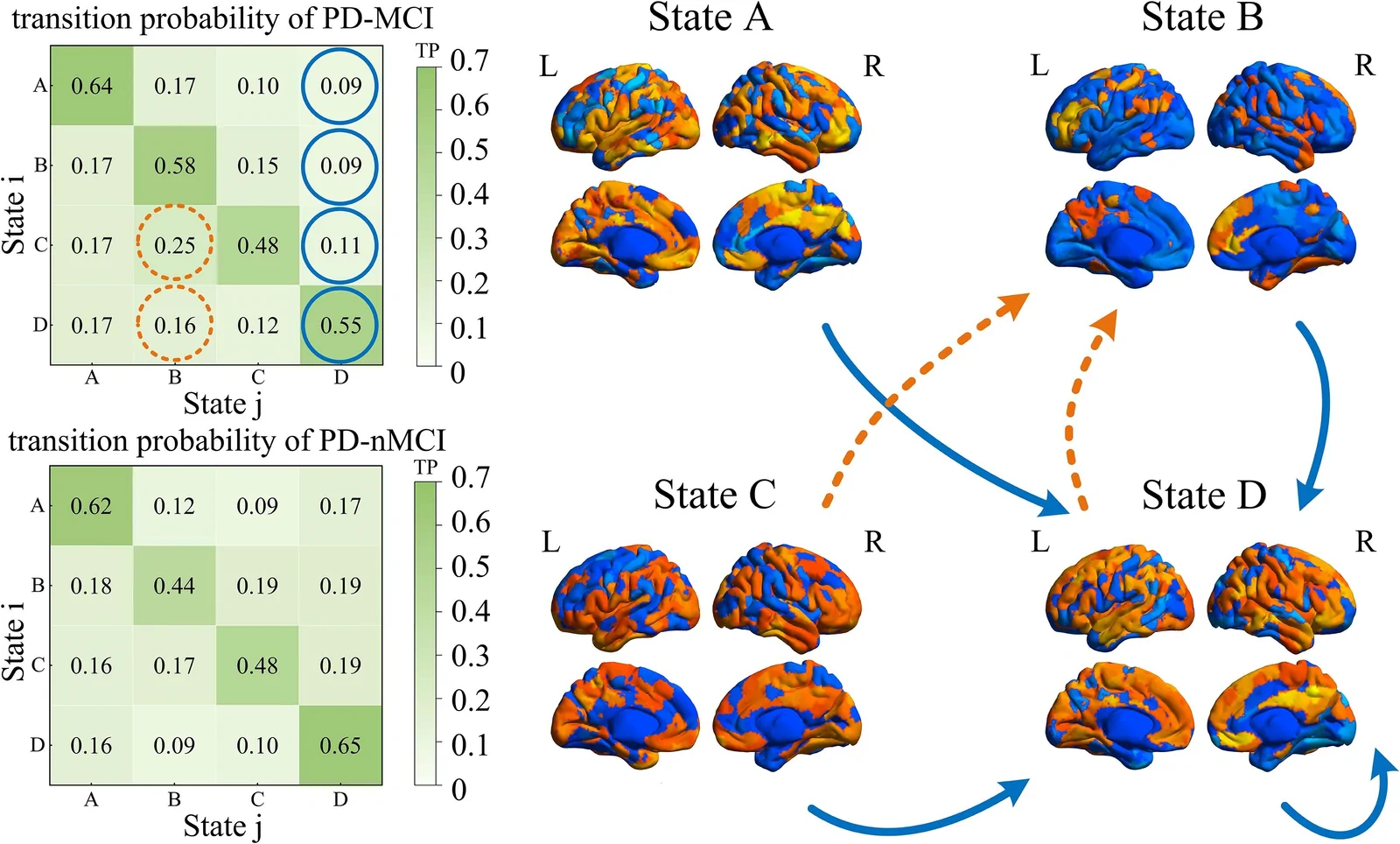
**Comparison of transition probabilities of brain functional status between Parkinson’s disease with MCI (N = 13) and Parkinson’s disease with nMCI groups (N = 16).** The dashed arrow represents a significant increase in state transition probability in the Parkinson’s disease with MCI group compared to the Parkinson’s disease with nMCI group (independent sample *t*-test: C-B, *P* = 0.040, *t* = 2.157; D-B, *P* = 0.014, *t* = 2.616). The solid line arrow indicates a significant decrease in state transition probability in the PD-MCI group (A-D, *P* = 0.031, *t* = −2.281; B-D, *P* = 0.004, *t* = −3.199; C-D, *P* = 0.019, *t* = −2.488; D-D, *P* = 0.037, *t* = −2.192). MCI, mild cognitive impairment; nMCI, without cognitive impairment; TP, transition probability. Detailed data are shown in Table 2.

Furthermore, Spearman correlation analysis revealed that the transition probabilities from states A, B, and C to state D were significantly positively correlated with MoCA scores (*r* = 0.433, *P* = 0.019; *r* = 0.472, *P* = 0.010; *r* = 0.505, *P* = 0.005). As shown in Fig. 6, the probability of transitioning into state D progressively decreased with declining clinical cognitive performance, as reflected by lower MoCA scores. Given that state D is considered a cognitively integrative state, a reduced transition to this state may hinder the effective allocation of neural resources necessary for cognitive processing, aligning with the observed deterioration in clinical cognitive function among Parkinson’s disease patients. Notably, state D exhibited both higher state occupancy rate and state duration in the Parkinson’s disease with nMCI group but was significantly reduced in the Parkinson’s disease with the MCI group. This further supports the notion that state D plays a crucial role in maintaining normal cognitive function, and its diminished temporal expression and kinetic accessibility may serve as an early marker of cognitive impairment in Parkinson’s disease.

**Figure 6:**
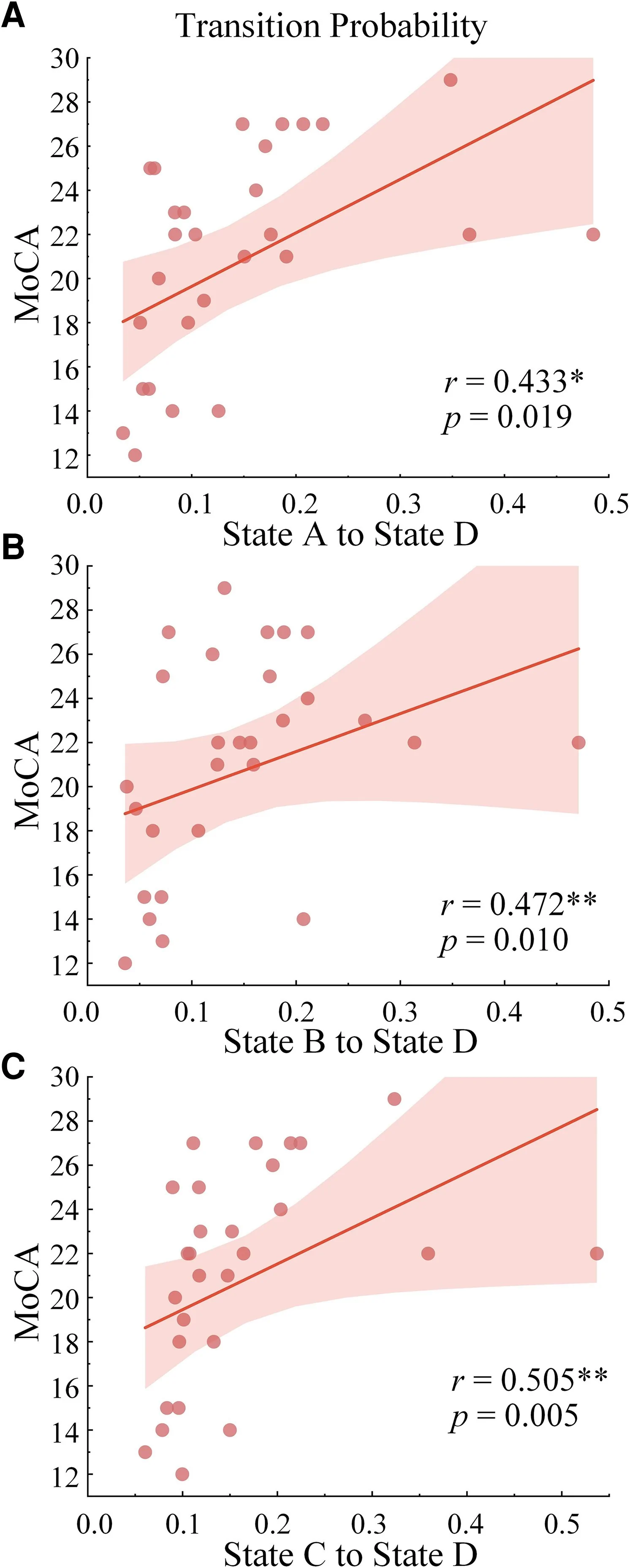
**The Spearman correlation between brain functional state transition probability and the MoCA scale.** * For *P* < 0.05, and ** for *P* < 0.01. Each data point represents the MoCA score and corresponding transition probability for one patient (*N* = 29). (**A**) Scatter plot showing the positive correlation between the transition probability from State A to State D and MoCA scores (*r* = 0.433, *P* = 0.019). (**B**) Scatter plot showing the positive correlation between the transition probability from state B to state D and MoCA scores (*r* = 0.472, *P* = 0.010). (**C**) Scatter plot showing the positive correlation between the transition probability from state C to state D and MoCA scores (*r* = 0.505, *P* = 0.005). MoCA, Montreal Cognitive Assessment.

## Discussion

In this study, we investigated the dynamic reconfiguration of brain functional states in Parkinson’s disease patients with and without MCI by applying an advanced LEiDA framework to source-reconstructed cortical signals. Through the extraction of instantaneous phase-locking functional connectivity patterns, we identified four recurrent brain states. Our analysis revealed distinct alterations in both the temporal properties and transition dynamics of these states in patients with Parkinson’s disease with MCI. These findings provide novel insights into the neural mechanisms subserving cognitive decline in Parkinson’s disease and suggest candidate dynamic biomarkers for clinical translation.

### Altered temporal characteristics in Parkinson’s disease with MCI

In the Parkinson’s disease with the MCI group, both the state occupancy rate and state duration of state B were significantly increased, whereas those of state D were markedly decreased, suggesting a bidirectional imbalance potentially driven by dopaminergic dysregulation and broader neuromodulatory failure. Crucially, the abnormally prolonged engagement in state B, characterized by the co-deactivation of the VN and LN, may impair the coordinated processing of visual and emotional information. Previous studies have linked structural and functional disconnection between these networks to cognitive decline in Parkinson’s disease.^40,41^ The increased prevalence of this hypoactive state in Parkinson’s disease with MCI patients suggests a loss of neural flexibility and a failure to recruit appropriate resources for task-ready cognition. Excessive and prolonged deactivation of these sensory-affective hubs represents an electrophysiological marker of cognitive impairment and network breakdown.^42^

Concurrently, the diminished expression of state D may reflect impaired top-down cognitive control, as this state is defined by the co-activation of the DMN and VAN.^43^ The DMN is heavily implicated in various cognitive processes and exhibits distinct activation patterns across different cognitive tasks.^44^ In Parkinson’s disease with MCI, we hypothesize that disrupted DMN-VAN coordination prevents effective switching between introspective and externally-oriented processes, resulting in inefficient allocation of attentional resources.^45^ This is further corroborated by the significant positive correlation between state D occupancy and MoCA scores, supporting its role as a cognitively integration state. Our findings provide further evidence in support of the seminal framework by Fox *et al*.,^46^ which highlighted the importance of inter-network coupling between the DMN and attentional systems for flexible cognitive resource deployment. Thus, the reduction of this state in Parkinson’s disease with MCI may thus reflect a breakdown in global network efficiency.

### Disrupted state transition dynamics and cognitive flexibility

Beyond static temporal metrics, analysis of state transitions revealed a disrupted dynamic landscape in Parkinson’s disease with MCI. Specifically, Parkinson’s disease with MCI patients exhibited significantly increased transition probabilities from states C and D into state B, coupled with decreased probabilities of transitioning to state D from any other state (A, B, or C). This transition pattern, marked by a hyper-convergence towards state B and a systemic attrition away from state D, suggests a loss of functional flexibility and impaired dynamic regulation. One possible mechanism is an LN-driven ‘attractor effect’, wherein disinhibition of the LN anchors the network into a dysfunctional trajectory, potentially trapping Parkinson’s disease with MCI patients in cognitively irrelevant processing loops.^47^ This may be driven by the progressive degeneration of key neuromodulatory systems, particularly the cholinergic and noradrenergic pathways, which are known to be severely affected in Parkinson’s disease with MCI and are critical for regulating network stability and cognitive flexibility.^48^

Dynamic decoupling between the salience network and the DMN may hinder the capacity for flexible network reconfiguration during task-related cognitive engagement.^45^ This imbalance in transition dynamics closely mirrors the clinical phenotype of Parkinson’s disease with MCI: the overexpression of state B may exacerbate susceptibility to task-irrelevant distractions (e.g. visually salient stimuli), whereas the underrepresentation of state D may compromise the maintenance of goal-directed behaviour. The observed trapping in state B and impaired access to state D may reflect a pathological impairment in large-scale network coordination, consistent with combined effects of limbic dysfunction, cholinergic denervation, and possible co-existing Alzheimer’s disease pathology (e.g. amyloid-β and tau protein), which collectively disrupt the brain's ability to transition efficiently between functional states required for cognition.^49,50^

Together, these temporal dynamic abnormalities not only deepen our understanding of the neural mechanisms underlying cognitive impairment in Parkinson’s disease but also provide a theoretical foundation for the development of diagnostic and therapeutic strategies that leverage state-dependent brain network dynamics.

### Implications for mechanisms and clinical applications

Collectively, our findings indicate that cognitive impairment in Parkinson’s disease is associated with a pathological reorganization of large-scale network dynamics, characterized by an overexpression of a functionally disconnected state (state B) and reduced accessibility of a cognitively supportive state (state D). These alterations are likely driven by underlying neurophysiological mechanisms, including dopaminergic depletion, cholinergic denervation, and large-scale network disintegration, resulting in increased network rigidity and impaired top-down regulation. Importantly, the state occupancy rate, state duration, and transition probabilities of these states may serve as neurophysiological biomarkers for early detection and longitudinal monitoring of cognitive decline in Parkinson’s disease.

From a clinical perspective, these dynamic network signatures open new avenues for targeted interventions. Neuromodulatory approaches, such as transcranial magnetic stimulation or cholinesterase inhibitors, could be designed to restore the dynamic balance between maladaptive and beneficial brain states, thereby potentially improving cognitive outcomes. Furthermore, the LEiDA-based framework provides a high-temporal-resolution alternative to traditional sliding-window analyses, enabling a more precise characterization of disease-specific dynamics and offering deeper insights into the network pathophysiology of Parkinson’s disease.

A limitation of the current study is the absence of an age-matched healthy control (HC) group. Nevertheless, the current study focuses primarily on distinct neurodynamic trajectories separating Parkinson’s disease with MCI from Parkinson’s disease with nMCI, rather than on distinguishing Parkinson’s disease pathology from HC. Consequently, while the absence of an age-matched HC group limits our ability to attribute the observed alterations exclusively to Parkinsonian pathology, it does not diminish the validity or interpretability of the primary findings regarding the distinct neurodynamic signatures that separate Parkinson’s disease with MCI from Parkinson’s disease with nMCI within the Parkinson’s disease population. The identified alterations in brain state dynamics remain clinically relevant as potential biomarkers for cognitive decline within Parkinson’s disease, which is the central investigative question of this work. Future studies incorporating HC cohorts will be valuable to further delineate the specificity of these dynamic patterns and to determine their relationship to normal ageing processes.

In conclusion, the present study elucidates the neurobiological mechanisms underlying cognitive decline in Parkinson’s disease, emphasizing the pivotal role of aberrant spatiotemporal dynamics in functional brain states. The persistent dominance of state B, likely reflecting limbic dysregulation and reduced neuromodulatory tone, combined with the inaccessibility of state D, which supports attention and cognitive integration, reflects a shift towards suboptimal and rigid network configurations. These findings could inform the development of dynamic biomarkers for the diagnosis, prognosis, and therapeutic targeting of Parkinson’s disease-associated cognitive impairment.

## Data Availability

The data collected in this study are available from the corresponding author, upon reasonable request. The MATLAB scripts used for data analysis in this study are deposited in a public GitHub repository (https://github.com/yuxin1203/Improved-LEiDA-Algorithm).
